# Comparative Mitogenome of Phylogenetic Relationships and Divergence Time Analysis within Potamanthidae (Insecta: Ephemeroptera)

**DOI:** 10.3390/insects15050357

**Published:** 2024-05-15

**Authors:** Zhi-Qiang Guo, Ya-Jie Gao, Yu-Xin Chen, Le-Mei Zhan, Kenneth B. Storey, Dan-Na Yu, Jia-Yong Zhang

**Affiliations:** 1 College of Life Sciences, Zhejiang Normal University, Jinhua 321004, China; 2 School of Bioengineering, Aksu Vocational Technical College, Aksu 843000, China; 3 Department of Biology, Carleton University, Ottawa, ON K1S 5B6, Canada; 4 Key Lab of Wildlife Biotechnology, Covnservation and Utilization of Zhejiang Province, Zhejiang Normal University, Jinhua 321004, China

**Keywords:** Potamanthidae, mitochondrial genome, phylogenetic relationship, selective stress analysis, divergence time

## Abstract

**Simple Summary:**

As one of the burrowing mayfly groups with large mandibular tusks, the phylogenetic relationships within Potamanthidae are always controversial. There are at least two opposite hypotheses for mayfly grouping: Potamanthidae + (Ephemeridae + Polymitarcyidae) and (Potamanthidae + Ephemeridae) + Polymitarcyidae. Because of the indeterminate origin time of this group, the present study aimed to reconstruct the phylogenetic relationship and explore the origin time of Potamanthidae based on mitochondrial (mt) genomes. In addition, the protein-coding genes (PCGs) of these mt genomes may undergo positive selection when these species live in low-temperature environments.

**Abstract:**

Potamanthidae belongs to the superfamily Ephemeroidea but has no complete mt genome released in the NCBI (except for two unchecked and one partial mt genome). Since the sister clade to Potamanthidae has always been controversial, we sequenced seven mt genomes of Potamanthidae (two species from *Rhoenanthus* and five species from *Potamanthus*) in order to rebuild the phylogenetic relationships of Potamanthidae in this study. The divergence time of Potamanthidae was also investigated by utilizing five fossil calibration points because of the indeterminate origin time. In addition, because *Rhoenanthus coreanus* and *Potamanthus luteus* are always in low-temperature environments, we aimed to explore whether these two species were under positive selection at the mt genome level. Amongst the 13 PCGs, CGA was used as the start codon in COX1, whereas other genes conformed to initiating with an ATN start codon. From this analysis, UUA (L), AUU (I), and UUU (F) had the highest usage. Furthermore, the DHU arm was absent in the secondary structure of S1 in all species. By combining the 13 PCGs and 2 rRNAs, we reconstructed the phylogenetic relationship of Potamanthidae within Ephemeroptera. The monophyly of Potamanthidae and the monophyly of *Rhoenanthus* and *Potamanthus* were supported in the results. The phylogenetic relationship of Potamanthidae + (Ephemeridae + Polymitarcyidae) was also recovered with a high prior probability. The divergence times of Potamanthidae were traced to be 90.44 Mya (95% HPD, 62.80–121.74 Mya), and the divergence times of *Rhoenanthus* and *Potamanthus* originated at approximately 64.77 Mya (95% HPD, 43.82–88.68 Mya), thus belonging to the late Pliocene Epoch or early Miocene Epoch. In addition, the data indicated that *R. coreanus* was under negative selection and that ATP8 and ND2 in Potamanthidae had a high evolutionary rate.

## 1. Introduction

Ephemeroptera comprises 42 families, under 500 genera, and nearly 4000 species [1,2]. Widely distributed in freshwater or brackish water habitats, mayflies are found on all continents, and some species even existed in the Nearctic region. They are known for their short-lived imago stage, which in some cases lasts only for several minutes [3,4]. They display a unique and primitive method of development called prometabola development, which has four stages (egg, nymph, subimago, and imago), and they are the only insects that need to molt once to enact their mating ability after forming wings [5,6]. Potamanthidae (commonly called hacklegills) belong to the superfamily Ephemeroidea, and they are mainly distributed throughout the Palaearctic and Oriental regions. At present, 32 species of Potamanthidae have been recorded in the world [7,8,9]. The nymph of Potamanthidae feeds mainly on humus residue and structures that allow it to adapt to a flowing water environment, such as large mandibular tusks for filter feeding and feather gills for breathing [10,11]. Compared with other families in existing mayflies, Potamanthidae, Ephemeridae, Polymitarcyidae, Ichthybotidae, Euthyplociidae, and Palingeniidae all have mandibular tusks, and for some species in Ephemerellidae and Leptophlebiidae, it was also found that mandibular tusks and the evolution of tusks have always been unknown [12,13,14,15,16].

Pictet first established the genus *Potamanthus* in 1843, but for ambiguous reasons, some species belonging to Leptophlebiidae and Ephemerellidae were misplaced in this genus [17]. *Potamanthodes* was classified as a subgenus of *Potamanthus* by Bae and McCafferty in 1991 [4], whereas Kluge put *Potamanthodes* into *Rhoenanthus* [18]. Studies by Kluge supported the paraphyletic relationship in Potamanthidae and Euthyplociidae [18]. Li and Zhou redefined the phylogenetic relationships of Potamanthidae by regarding *Potamanthodes* as an independent genus in 2022 [4,19]. At present, four genera, including *Anthopotamus, Potamanthus, Potamanthodes,* and *Rhoenanthus*, have been reported around the world. The monophyly of Potamanthidae has been supported by many researchers, but the phylogenetic relationship of its sister clade was always controversial. Edmunds suggested that the sister clade of Potamanthidae was Euthyplociidae [20], whereas McCafferty supposed that Potamanthidae was the sister clade of all burrowing mayflies except Behningiidae [21]. In 2005 and 2009, Odgen et al. found that Potamanthidae was the sister clade to the remaining burrowing mayflies and some species of Pannota [22,23]. Thereafter, based on over 400 targeted genomic protein coding regions, Odgen et al. suggested that Potamanthidae was the sister clade of all remaining burrowing mayflies [24], and this result was consistent with the work of Miller et al. [14]. In recent years, Wang et al. supported the phylogenetic relationship of Potamanthidae + (Ephemeridae + Polymitarcyidae), whereas Tong et al. suggested the phylogenetic relationship of (Potamanthidae + Ephemeridae) + Polymitarcyidae [25,26]. Of note, most researchers have been involved in morphological studies and other aspects of Potamanthidae rather than molecular research [11,27,28,29,30,31].

The earliest mayfly fossils that have been found date back to the Carboniferous period, and subsequently, Kluge proposed that these species were more closely related to Thysanura rather than being true mayflies [32,33]. The oldest fossil mayflies were discovered in the Early Permian strata of Moravia, Slovakia [34]. Most studies showed that Ephemeroptera dated from the Late Carboniferous or Early Permian and had their highest species richness during the Mesozoic Era. It was believed that the glacial activities during the Pleistocene Epoch were significant periods for speciation [35,36,37], but research on the divergence times at the family level within the Ephemeroptera are relatively limited. Back in the early 19th century, researchers began to concentrate on the fossils of Ephemeroptera, and significant progress had been made in the study of these fossils, with research now encompassing not only morphological descriptions but also aspects such as geographical distribution and systematics. Information on a few Potamanthidae fossils is available online (www.fossilworks.org), being focused on *Olindinella gracilis*, which has already gone extinct [38], and *Nanophemera myanmarensis,* and *Crepotamanthus spinitarsus*, which are possibly closely related to Potamanthidae and have also gone extinct [30,31]. Based on 1478 single-copy genes, Misof et al. proposed that about 239 Mya, mayfly began to diversify [39]. Tong et al. first assessed the divergence time within Ephemeroptera using four fossil calibration points and determined that Potamanthidae originated in the Cretaceous period, about 105.04 Mya [40]. And then, García-Girón et al. calculated that the Potamanthidae diverged approximately 106.03 Mya [29].

Known as the “energy factories” in eukaryotes, mitochondria are semi-autonomous organelles that are closely involved in energy metabolism, providing more than 95% of the ATP energy needed for vital activities via oxidative phosphorylation (OXPHOS) [41,42]. Since the first mayfly mitochondrial (mt) genome of *Parafronurus youi* was released [43], there have been more and more sequenced mayfly mt genomes. Insect mt genomes can range in size from 14 to 20 kb and are usually double-stranded circular molecules that encode 37 canonical genes, including 13 protein-coding proteins (PCGs), which are vital for the electron transfer chain, 2 ribosomal RNAs (rRNA), 22 transfer RNAs (tRNAs) for transferring PCGs, and 1 control region (CR), which is also called the A-T-rich region [44,45,46]. Because of its rapid evolutionary rate, matrilineal inheritance, and small molecular size, the mt genome is quite useful in reconstructing the phylogenetic relationships among Insecta [47,48,49,50,51,52,53]. Up until 15 January 2024, there were no complete mt genomes of Potamanthidae (except two unchecked and one partial mt genome) available on the NCBI website (https://www.ncbi.nlm.nih.gov, accessed on 15 January 2024).

Adaptive evolution, regarded as one of the most important strategies for survival and continuation of organisms, involves key processes that allow organisms to adapt to different environments. As part of this adaptation, both their morphology and living habits may undergo changes to fit new conditions [54,55]. The mt genome always seems to be a rigorous neutral marker of adaptation [56], but some researches have pointed out that the mt genome is under positive selection, being associated with a variable environment [57]. In Ephemeroptera, Xu et al. explored the Heptageniidae branch, living at low temperatures, as the foreground branch and found that there were 27 positive selection sites distributed in COX1, Cyt b, ND1, ND2, ND3, ND4, ND5, and ND6 [57]. Yang et al. found that the primitive species in Pterygota would increase their energy metabolism during flight when the 13 PCGs were under positive selection and that indirect flight insects were under stronger positive selection than direct insects [58]. Li et al. identified significant positive selection acting on ATP6, ND2, ND3, ND4, and ND5 in grasshoppers inhabiting the Tibetan Plateau [59]. Moreover, Yuan et al. compared the *Gynaephora* species living at high altitudes with those species living at low altitudes and found that the former had five positive selection sites detected in ND5 [60].

In the present study, we sequenced two mt genomes of Potamanthidae from Dandong, Liaoning Province in China, which has an annual average temperature of 8.8 °C (https://www.dandong.gov.cn/html/DDSZF/202202/0164543540781797.html), as well as five mt genomes we obtained of Podamathidae from Fujian in the Yunnan and Zhejiang provinces in China. We combined mt genomes acquired from the NCBI with our newly sequenced mt genomes to explore the following: (1) an analysis of the characteristics of mt genomes in Potamanthidae; (2) a reconstruction of the phylogenetic relationship of Potamanthidae within Ephemeroptera and calculation of the divergence times of each clade within Ephemeroptera; and (3) determination of whether the mt genomes of *R. coreanus* and *P. luteus* collected from Dandong were under positive selection or not during adaption to low temperatures.

## 2. Materials and Methods

### 2.1. Sampling and DNA Extraction

Between 2022 and 2023, we collected seven species of Potamanthidae (all in the larval stage) using D-frame aquatic nets. The samples were preserved in 100% alcohol and stored at −20 °C in Zhang’s lab at the College of Life Sciences of Zhejiang Normal University in Zhejiang, China. Based on the morphological characteristics, we identified the species under an SMZ-1500 stereomicroscope (Nikon, Tokyo, Japan). Detailed information on the sampling localities is shown in Table 1. Whole individuals were used for extraction of the total genomic DNA using an Ezup Column Animal Genomic DNA Purification Kit (Sangon Biotech Company, Shanghai, China).

### 2.2. Sequencing and mt Genome Assembling

We isolated the total genomic DNA by using a kit from Infinite 200-PRO (Tecan, Grödig, Austria) and sent the samples with a concentration exceeding 500 ng/mL to BGI Tech Inc. (Shenzhen, China) for next-generation sequencing. The raw sequence data were obtained using the Illumina HiSeq 2000 platform to acquire clean frontend and backend data in the file format of FASTQ. Then, the SOAPnuke platform was used to filter low-quality adapter contamination and reads containing high “N” bases. Secondary quality control was performed through comparative analysis of the DNA genomes, RNA genomes, and rRNA sequences. To extract the mt genomes and ensure the credibility of the results, we compared the results using NOVOPlasty v.4.2 [61], GetOrganelle v.1.7.1 [62], and MitoZ [63] to obtain the final mt genomes.

### 2.3. mtDNA Annotation and Structural Analysis

The online MITOS2 service (http://mitos2.bioinf.uni-leipzig.de/index.py, accessed on 15 October 2023) [64] and tRNAScan-SE 2.0 (lowelab.ucsc.edu/tRNAscan-SE, accessed on 16 October 2023) [65] were used to identify tRNA genes. Using the start codons, stop codons, and the length of the 13 PCGs of Ephemeroptera published in the NCBI, we annotated the 13 PCGs of 7 mt genomes, referencing the *Potamanthus* sp. MT-2014 KM244674. To ensure the correctness of the annotation, we translated the 13 PCGs with Mega11 using invertebrate codons. The 12S rRNA (12S) and 16S rRNA (16S) were annotated by Clustal W alignment in Mega11 [66]. The GenBank accession numbers of the seven samples are shown in Table 1. The circular maps of the mt genomes were drawn using CG View v1.0 [67], and the secondary structures of each were drawn online (rna.tbi.univie.ac.at/forna/, accessed on January 16, 2024). Nucleotide compositional bias was assessed using the A/T skew ((A − T)/(A + T)) and GC skew ((G − C)/(G + C)) [68]. PhyloSuite v.1.2.3 [69] was employed to analyze the amino acid usage and relative synonymous codon usage (RSCU) of each mt genome.

### 2.4. Phylogenetic Analyses

Eighty mt genomes were used for phylogenetic relationship analyses, including the 7 mt genomes newly reported in this paper, and 73 mt genomes downloaded from the NCBI for Ephemeroptera in 15 families (Baetidae, Heptageniidae, Neoephemeridae, Leptophlebiidae, Isonychiidae, Ephemerellidae, Ephemeridae, Viemamellidae, Potamanthidae, Caenidae, Siphluriscidae, Polymitarcyidae, Ameletidae, Siphlonuridae, and Teloganodidae) [26,43,51,70,71,72,73,74,75,76,77,78,79,80,81,82,83]. Siphluriscidae is typically regarded as occupying a basal position within the Ephemeroptera [82,84]. Hence, we designated *Siphluriscus chinensis* (HQ875717 and ON729391) as the outgroup in this study. A detailed list of all mt genomes that were utilized is presented in Table S1. We extracted the 13 PCGs and 2 rRNAs using PhyloSuite v.1.2.3 [69] and aligned them with MAFFT v7.475 [85]. Subsequently, Gblocks 0.91b was applied to select the conserved regions [86]. PhyloSuite v.1.2.3 was then used to concatenate the 13 PCGs and 2 rRNAs to the maximum data matrix. We leveraged PartionFinder 2.2.1 in Python software packages to find the optimal model [87]. Based on the maximum dataset, 15 subset partitions with different models were recognized, and their respective results are displayed in Table 2. AliGROOVE v.107 [88] was used to detect the heterogeneity of 80 mt genomes, and we checked the topological structures using four-cluster likelihood mapping (FcLM) analysis with IQtree v.1.6.12 [89].

We reconstructed the phylogenetic relationship of Ephemeroptera using two different programs for creating phylogenetic trees: Bayesian inference (BI) and maximum likelihood (ML), respectively. RaxML v.8.2 was based on the above partition schemes, and a total of 1000 runs were performed with the bootstrap value set to 100 [90]. For BI analysis, we used MrBayes 3.2 based on Markov Chain Monte Carlo [91]. Starting from a random tree, the program conducted 10 million generations, during which trees were sampled and saved every 100 generations (after the first 25% were discarded). Finally, the phylogenetic trees obtained were visualized and beautified using FigTree v.1.4.4 [92] and Afinity Designer 2 [93], respectively.

### 2.5. Divergence Time Analysis

Derived from the phylogenetic tree, the divergence time of Potamanthidae was evaluated with MCMCTree using the PAML 4.8 package [94]. Due to the current scarcity of fossil groups within Ephemeroptera and the fact that most fossils cannot be associated with Euplectoptera, this study selected fossils that could correspond with Euplectoptera classification groups as fossil calibration points for estimating divergence times. In this study, we gathered five time points of fossil formation as the calibration point for calculating the divergence time: one from the web (www.fossilworks.org) and four from literature reports [95,96,97,98]. As a consequence, the accurate time of fossil formation could not be predicted, and thus the minimum and maximum ages were used for the evaluation. The first fossil calibration point we selected was Siphlonuridae (159–160.6 Mya) in the middle Jurassic period [96]. The second and third calibration points chosen were the recorded fossils of Vietnamellidae (98.17–99.41 Mya) and Atalophlebiinae of Leptophlebiidae (15–20 Mya) [95,98]. The formation time of *Neophemera* (48.6–55.8 Mya) gathered from the web was recognized as the fourth fossil calibration point for the family Neoephemeridae, and the final fossil calibration point was *Ephemerella* of Ephemerellidae (41.3–47.8 Mya) [97]. We set the root age at 239 Mya in the Mesozoic Era [39]. After calculating the substitution rate using the subprogram Baseml within the PAML software package, this information was then set to calculate the branch lengths based on the maximum likelihood method. Finally, we calculated the divergence time with a burn-in period of 1,000,000, sample frequency of 1000, and number of samples of 10,000. By using Tracer v.1.7.1 software [99] to check the ESS value with an mcmc.txt file, we accepted that convergence had been achieved when the final value was above 200. The final generated FigTree.tre file was visualized using FigTree v 1.4.4 [92].

### 2.6. Positive Selection Analysis

*P. luteus* and *R. coreanus* were collected from Dandong in Liaoning Province. These two species perennially inhabit cold environments. We used the site model, branch model, and branch-site model software from EasyCodeML v1.41 [100] to evaluate whether these two species that were long exposed to low temperatures were under positive selection. Based on the likelihood ratio tests (LRTs) and contrasted with the one-ratio model and the two-ratio model in the branch model, we checked whether the foreground branch was under positive selection. The branch-site model allowed us to compare a null model (Model A_null_) which permitted both neutral selection and negative selection with positive selection along with a foreground branch (Model A). We utilized the site model to detect whether the selection pressures acting on different amino acid sites were homogeneous or not. The LRT and Bayesian Empirical Bayes (BEB) methods were applied to assess the posterior probabilities of the two models and select sites that were under positive selection. Since the site model did not require specification of the foreground branches, in this study, all species within the Potamanthidae family were utilized for site model analysis. Concurrently, in order to prevent the influence of clades in the background branch, the branch of Potamanthidae was isolated from the phylogenetic tree. Because the *R. coreanus* and *P. luteus* were all from Dandong, and in order to prevent mutual interference between the two species, two different topological structures of trees were employed in our study. Among them, we set *R. coreanus* as the foreground branch when used as the topological structure without *P. luteus* (Analysis One), and then we set *P. luteus* as the foreground branch when using the topological structure without *R. coreanus* (Analysis Two).

## 3. Results

### 3.1. mtDNA Structure Analysis

**Figure 1:**
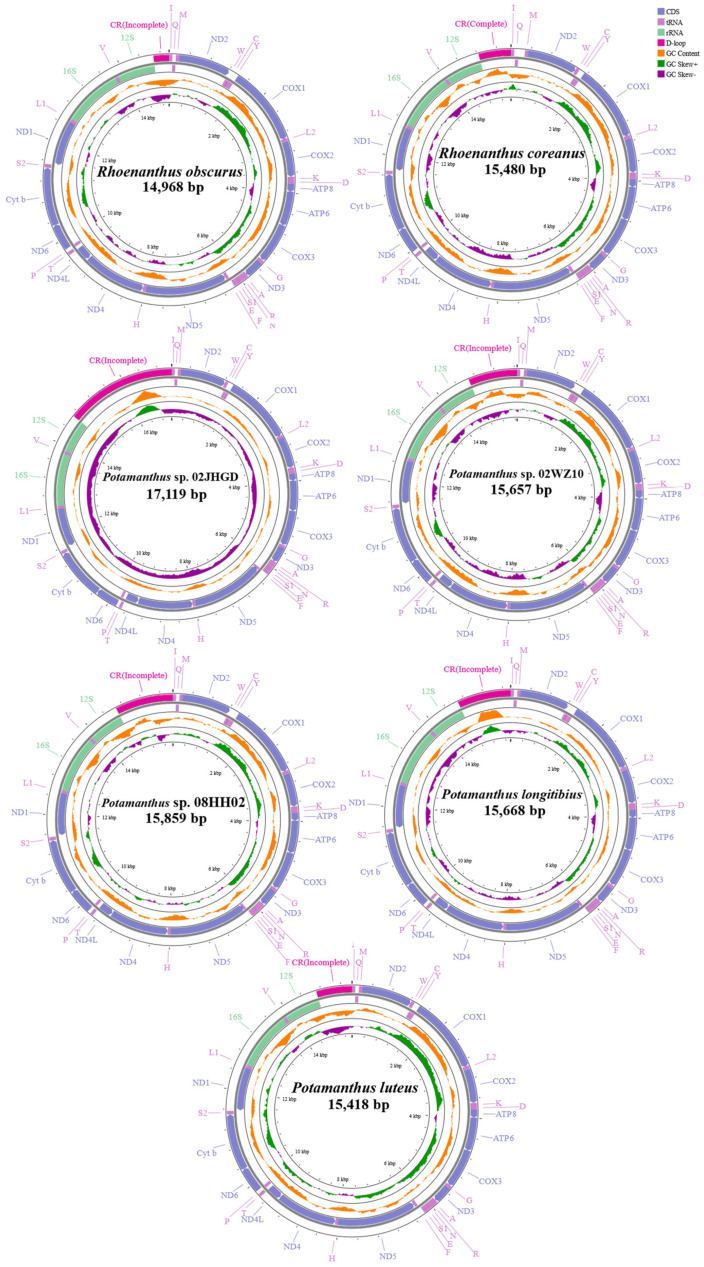
Circular maps of mitochondrial genes from seven species. Genes located on the outermost circle are those found on the heavy strand, and those found on the inner circle are on the light strand.

Seven mt genomes were obtained in this study, including one complete mt genome of *R. coreanus* with 15,480 bp and six nearly complete mt genomes that excluded a partial control region (CR). These seven genomes ranged in length from 14,968 bp for *R. obscurus* to 17,119 bp for *P.* sp. 02JHGD. All mt genome features conformed to the characteristics of insect mt genomes with 37 genes (13PCGs, 22 tRNAs, and 2 rRNAs) as well as a CR. The locations of features in the mt genomes of the seven species are shown in Table S2, and circular maps of the seven species are shown in Figure 1. We learned from the circular map that 23 genes (I, M, ND2, W, COX1, L2, COX2, K, D, ATP8, ATP6, COX3, G, ND3, A, R, N, S1, E, T, ND6, and Cyt b, S2) were positioned on the heavy strand, and 14 genes (Q, C, Y, F, ND5, H, ND4, ND4L, P, ND1, L1, 16S, V, and 12S) were positioned on the light strand.

Similar to other insect mt genomes, the AT content of the seven mt genomes exhibited a distinct bias, and the overall AT content ranged from 66.2% in *Potamanthus* sp. 02JHGD to 70.2% in *R. coreanus*. The AT content, CG skew, and AT skew of each species are shown in Table 3. For the usage rate of individual nucleobases in the seven mt genomes, we found that the percentage of each base showed a bias of T > A > C > G. Except for the COX1 gene, which started with CGA, all other PCGs of the seven mt genomes used the conventional invertebrate ATN (N = A/T/G/C) as the start codon. In terms of the stop codons used by the 13 PCGs, these were divided into complete codons (TAA and TAG) for most PCGs or incomplete codons (T and TA), which were particularly pronounced in COX1, COX2, COX3, ND5, and Cyt b.

**Figure 2:**
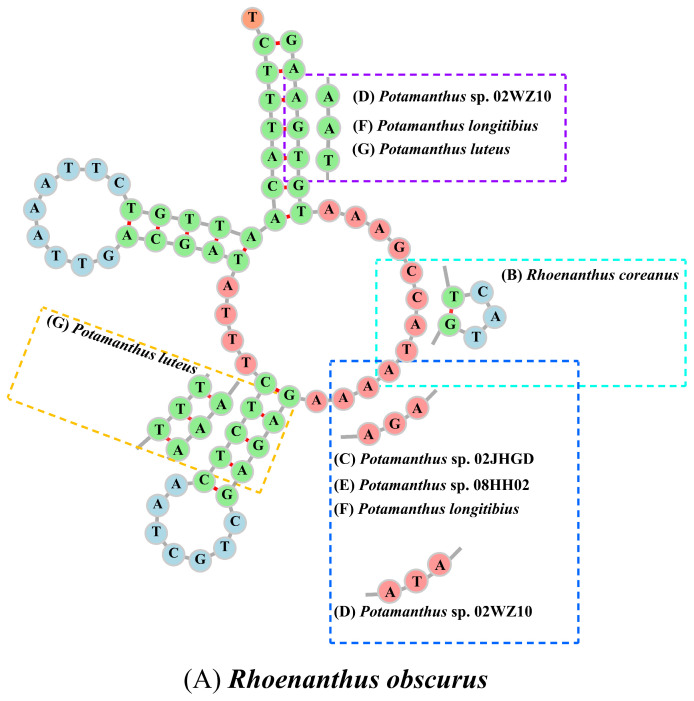
The secondary structures of S1 for each species.

**Figure 3:**
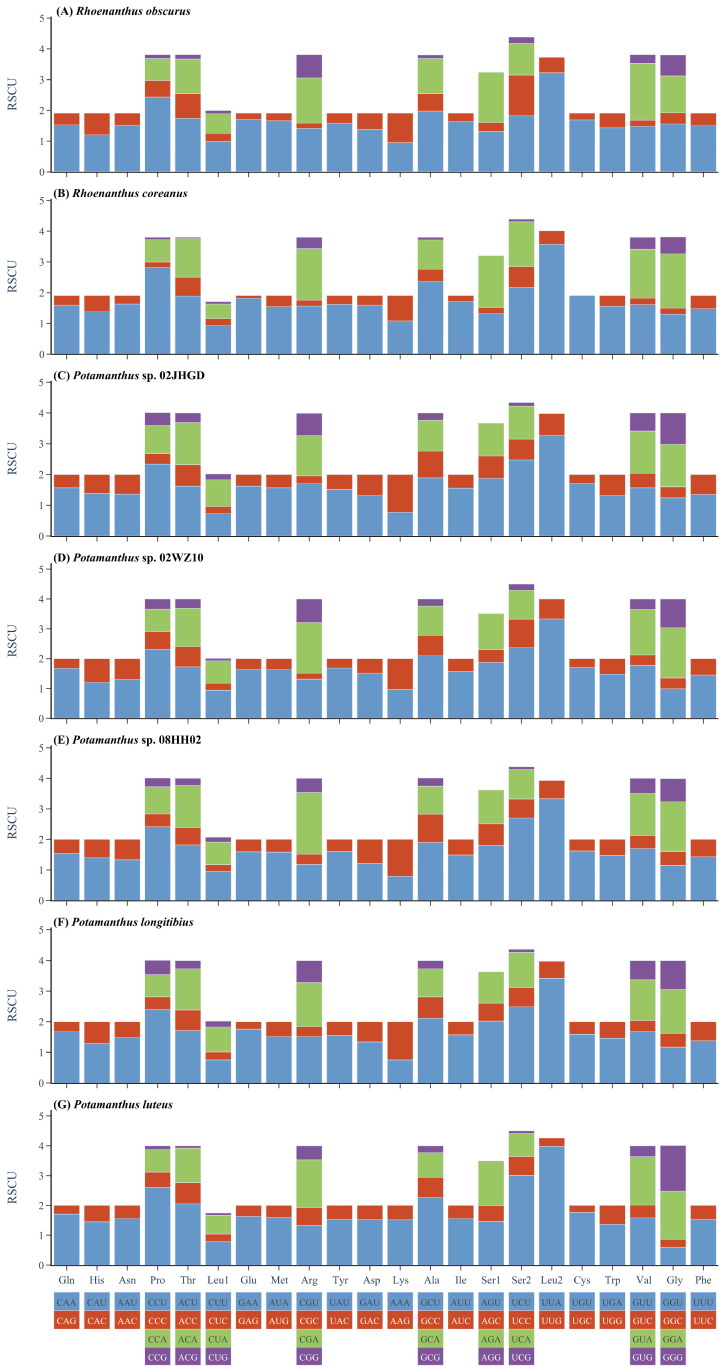
The relative synonymous codon usage of seven species of Potamanthidae.

All secondary structures of the tRNAs displayed the characteristic cloverleaf shape, except for the DHU arm in S1, which disappeared in seven species. The secondary structure of S1 in *R. obscurus* and the differences with other species are shown in Figure 2. And the secondary structures of all tRNAs from the seven species are shown in Figure S1. Among the seven mt genomes in this study, the codons with higher usage frequencies included AUA (I), AUU (I), and UUU (F), and the usage frequency of UUA (L) exceeded 300 in the seven species. As a result, Leu had the highest amino acid usage. The relative synonymous codon usage (RSCU) of the seven species is shown in Figure 3.

### 3.2. Phylogeny Analyses

**Figure 4:**
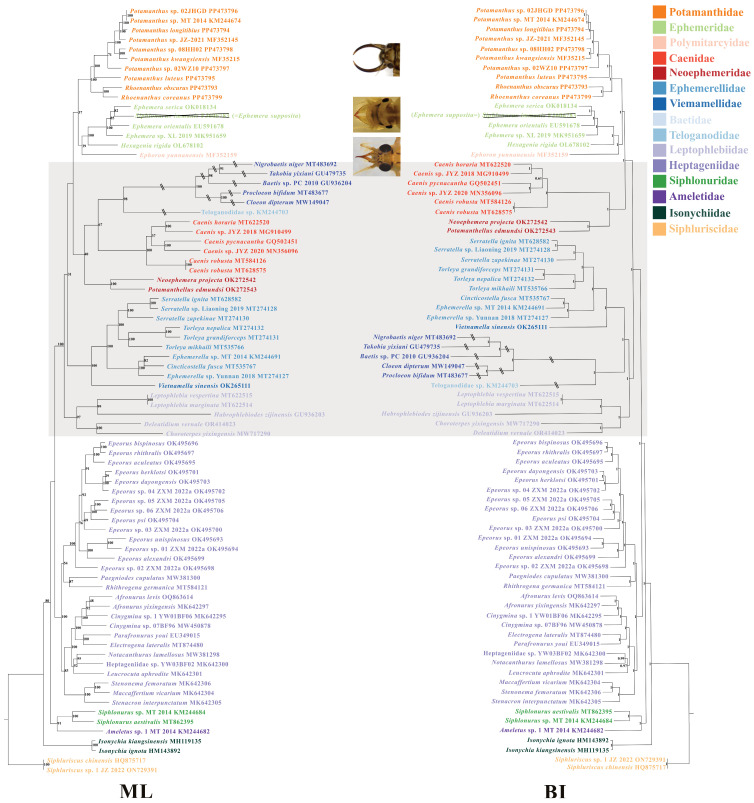
The ML tree (left) and BI tree (right) of all 80 Ephemeroptera species based on the 13 PCGs and 2 rRNAs. The out group consisted of *Siphluriscus chinensis* (ON729391 and HQ875717). All GenBank accession numbers are shown behind the species name. The numbers on the nodes show the bootstrap value and prior probability of the ML and BI tree, respectively. The shaded areas are topologically inconsistent areas. The photos used originated from other articles [12,13,101].

Two phylogenetic trees based on the dataset of 13 PCGs as well as 2 rRNAs were constructed using BI and ML analyses. Some differences in the topology of the two trees occurred and were focused mainly on the branches of Caenidae, Neoephemeridae, Baetidae + Teloganodidae, Ephemerellidae + Viemamellidae, and Leptophlebiidae (Figure 4). Owing to the FcLM analysis, this suggested that the phylogeny relationship of the ML tree had low scores (results shown in Figure S2) in addition to the high posterior probability of the BI tree. The following analysis focused mainly on the BI tree. We recovered the monophyly of all families in two trees, except Polymitarcyidae, Viemamellidae, Teloganodidae, and Ameletidae because of only one mt genome being used in each family. From the results, we found that Isonychiidae diverged next to Siphluriscidae with high support (posterior probability = 1, bootstrap value = 100). Both the monophyly of Potamanthidae and the relationship of Potamanthidae + (Ephemeridae + Polymitarcyidae) were recovered. At the genus level, we also recovered the monophyly of *Potamanthus* and *Rhoenanthus* with high support (posterior probability = 1, bootstrap value = 100). Teloganodidae appeared as a sister clade to Baetidae, but this was likely due to long branch attraction.

**Figure 5:**
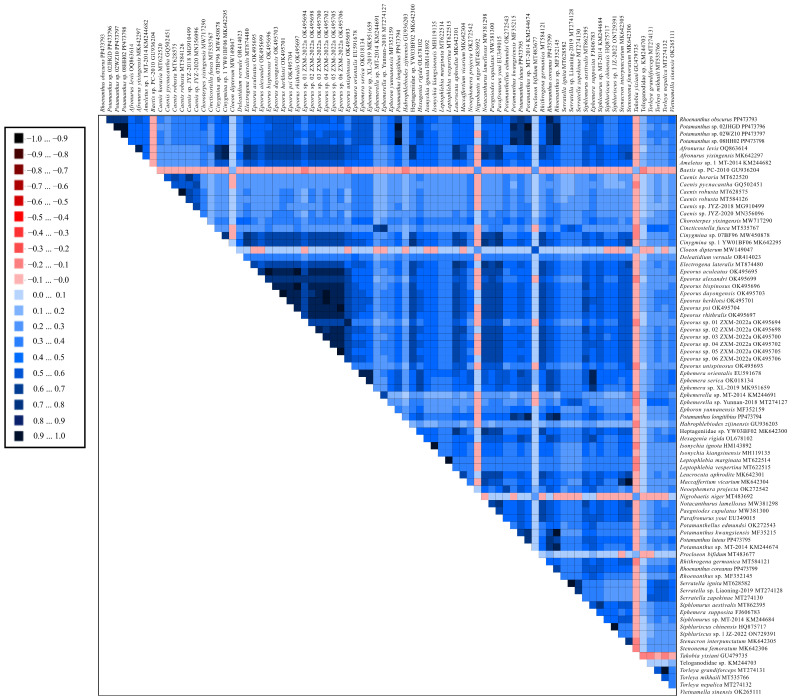
Analysis of heterogeneity in the 80 Ephemeroptera species based on the dataset of 13 PCGs and 2 rRNAs.

Moreover, long-branch attraction (LBA) was found in Teloganodidae and Baetidae, and the result of heterogeneity is shown in Figure 5. As shown in the figure, we could see high heterogeneity in five species belonging to Baetidae, and this heterogeneity could potentially be linked to phylogenetic long-branch attraction (LBA). LBA commonly results in the misinterpretation of evolutionary relationships, particularly when closely related lineages exhibit significantly different divergent rates of evolution.

### 3.3. Analysis of Divergence Time

**Figure 6:**
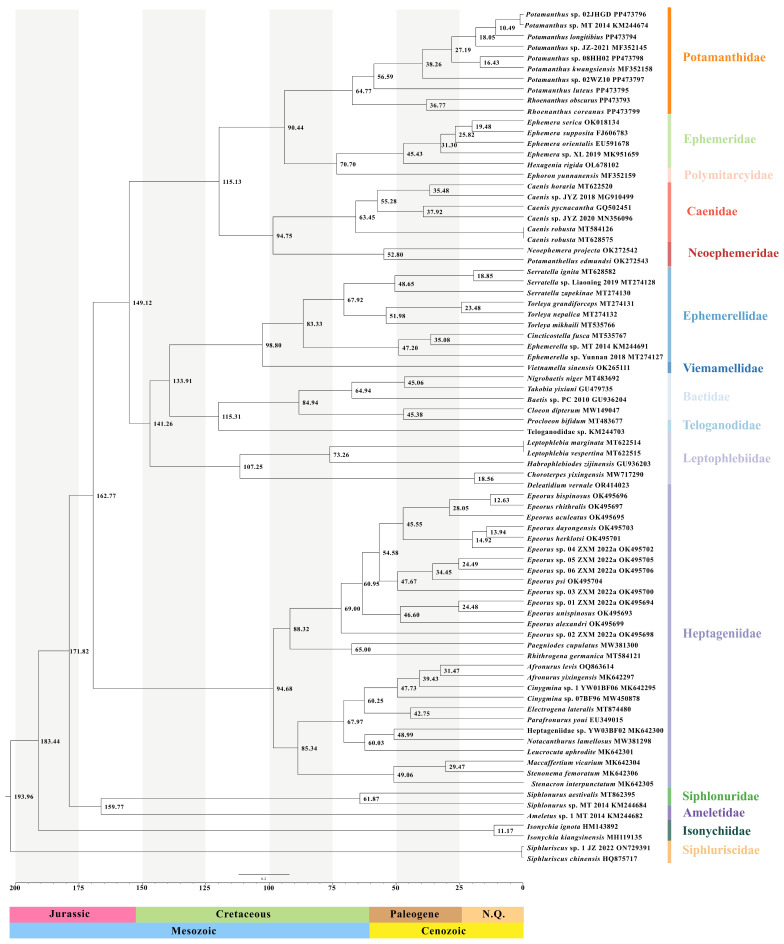
Divergence times within Ephemeroptera based on the phylogenetic tree and five fossil calibration points. The numbers above the nodes show the median ages.

The topology of the phylogenetic tree was used to conduct calculations of the divergence times. The results (Figure 6) suggest that Potamanthidae and Polymitarcyidae + Ephemeridae originated at 90.44 Mya (95% HPD, 62.80–121.74 Mya), including the early-to-middle Cretaceous period. Then, *Rhoenanthus* and *Potamanthus* diverged at 64.77 Mya (95% HPD, 43.82–88.68 Mya) in the late Pliocene Epoch or early Miocene Epoch. Analysis of *R. obscurus* indicated divergence at 36.77 Mya (95% HPD, 14.95–59.33). From these results, we found that Siphluriscidae was rooted at 193.96 Mya (95% HPD, 169.80–226.13 Mya), which covered parts of the Jurassic and Cretaceous geological epochs. The Isonychiidae that diverged next to Siphluriscidae originated at approximately 183.44 Mya (95% HPD, 167.02–202.64 Mya) and fell within the middle-to-late Jurassic period of the geological timescale. Furthermore, the final tree pointed out that Heptageniidae originated 162.77 Mya (95% HPD, 146.40–180.14 Mya) later than Siphluriscidae and Isonychiidae, and Caenidae diverged at 115.13 Mya (95% HPD, 86.11–145.70 Mya). Our results also show the divergence times between genera. For example, *Epeorus* diverged at 88.32 Mya (95% HPD, 63.29–114.59 Mya). The detailed divergence times for each family are presented in Table S3.

### 3.4. Analysis of Positive Selection

In the site model, 3699 amino acid sites from the 13 PCGs of 10 species of Potamanthidae were analyzed. When we compared Model 7 with Model 8 in the site model, we found that the *LRT p* values were extremely significant (*p* < 0.01), and the values of the BEB method at the 258th site located at ATP8 and the 2265th site located at ND2 were both greater than 0.90 (results shown in Table S4).

For the remaining data, two different topological structures were used for analysis. Analysis One excluded *P. luteus* and set *R. coreanus* as the foreground branch. This showed an *LRT p* value of 0.002, and the value of ω was 0.020, which was less than one. This result indicates that the branch of *R. coreanus* was under negative selection. Using the branch-site model, the *LRT p* value was also insignificant (*p* = 0.345), meaning that there was no site under positive selection. The results of the branch model and branch-site model are shown in Table S5 and Table S6, respectively.

Analysis two was performed without *R. coreanus* and set *P. luteus* as the foreground branch, with results indicating that the *LRT p* value was 0.859, which was more than the 0.05 in the branch model (results shown in Table S5). This indicates that there was no significant difference between *P. luteus* and the background branches. In the branch-site model, when we compared Model A with Model A null, we found the *LRT p* value was highly significant (*p* = 0.104), showing that there were no sites under positive selection (results are shown in Table S6).

## 4. Discussion

### 4.1. The Composition of mt Genomes

In this study, we obtained seven mt genomes, all with a circular double-stranded shape. In comparison with the mitochondrial genomes of other members of Ephemeroptera, all seven mt genomes of Potamanthidae conformed to their common characteristics. The main difference noted was in sequence length, with a greater length for the control region, which is the main non-coding region for replication and transcription and is known to have the fastest evolutionary rate and highest variation in the mt genome [102]. In an ideal situation, the frequency of usage for each synonymous codon would be equal [103]. However, numerous studies have shown that the usage of synonymous codons is a non-random process, exhibiting a phenomenon of unequal usage. In the seven species, the usage rates of the four codons UUA (L), AUU (I), AUA (M), and UUU (F) were all the highest and the same as the other species used in this study. For insect mt genomes, researchers found that dipteran insects tended to use codons ending with G or C [104,105], whereas insects of Hymenoptera tended to use codons ending with A or U [106]. Therefore, we inferred that in the mt genome of Ephemeroptera, protein-coding genes tended to use codons ending with A and U. Hecht used *E. coli* as a model organism to compare the translation efficiencies of 64 start codons, showing that AUG (M), GUG (V), and UUG (L) had the best translation efficiencies, whereas the translation efficiency of CGA (R) ranked behind most codons [107]. Interestingly, the start codon of COX1 in seven species was CGA, which rarely occurs in other groups but has been found in *Electrogena lateralis*, *Rhithrogena germanica*, and several other species in Ephemeroptera [57].

The secondary structure of the tRNA was cloverleaf-shaped, consisting of the acceptor arm, dihydrouracil (DHU) loop, anticodon loop, extra loop, and other components. In this study, we found that the DHU arms of S1 were absent in all seven species. This absence has also been observed in other insects, such as *Choroterpes yixingensis* (Ephemeroptera, Leptophlebiidae) and *Lopaphus albopunctatus* (Phasmida, Lonchodidae) [48,80].

### 4.2. The Phylogeny of Potamanthidae

We constructed BI and ML trees based on the Bayesian inference and maximum likelihood methods, respectively. The topologies of the ML tree and BI tree showed differences, as was also reported by Zhang et al. [108]. Early scholars supported the results of the BI tree (such as the one we constructed), whereas modern scholars are more inclined to use the results of the ML tree [18,22,23,40,57,79]. However, both the value of confidence and the results of FcML analysis point to the BI tree being a better result in the present study. Except for Siphlonuridae and Ephemeridae, the two trees recovered the monophyly of the remaining families, because *Siphlonurus immanis* (FJ606783) was assigned to Ephemeridae, which was consistent with many studies [57,73,79,80,81], and Yu et al. suggested that *S*. *immanis* might originally belong to the family Ephemeridae rather than the family Siphlonuridae [81]. We blasted the COI gene of *S*. *immanis* (FJ606783) in the BOLD dataset (http://www.boldsystems.org/index.php/IDS_IdentificationRequest, 9 May 2024) and found this species to belong to *Ephemera supposita*. Therefore, we suggest that the monophyly of Ephemeridae and the relationship of Potamanthidae + (Ephemeridae + Polymitarcyidae) were all recovered in this study. Both trees supported that Isonychiidae diverged next to Siphluriscidae, a result that was also supported by Cao et al. and Xu et al. in reconstructing the phylogenetic relationships of Ephemeroptera based on the 13 PCGs [76,109], but Baetidae was supported as the sister clade to other families of mayflies, excluding Siphluriscidae [24]. Teloganodidae was clustered with Baetidae, which was consistent with other research [26,57,76,77], but this result may have been caused by the long branch attraction. Both trees recovered the monophyly of Potamanthidae and suggested a relationship of Potamanthidae + (Ephemeridae + Polymitarcyidae), which was consistent with Wang et al. [25]. Although a low bootstrap value existed in the ML tree, Whitfield et al. pointed out that a larger amount of data often causes low support when exploring the connections between higher taxonomic groups in ancient insects because of rapid radiation [110]. The results in this study clearly contradicted Odgen’s idea, which was supposed in 2005 and 2009 and suggested that Potamanthidae was the sister clade of remaining burrowing mayflies and Pannota species [22,23]. In addition, the BI tree showed that Potamanthidae + (Ephemeridae + Polymitarcyidae) was closed to the clade of Caenidae + Neoephemeridae, which was consistent with Kluge and Odgen et al. [18,24]. The phylogenetic relationship of Potamanthidae has been discussed in conjunction with the burrowing mayfly [14,18,21,23,24]. And from the morphological characteristics among Potamanthidae, Ephemeridae, and Polymitarcyidae, the mandibular tusks protrude as a form of teeth and might be related to their living environment, since all species belonging to these three families were of the burrowing type. In the burrowing mayflies, the presence of mandibular tusks could assist in performing simple digging actions and transporting small gravel and sand, and they might also possess functions for attack and defense [101]. Interestingly, among the burrowing mayflies, the species in Behningiidae do not have the mandibular tusks, and through ancestral state reconstruction, Miller et al. suggested that Behningiidae are believed to have descended from a tusk-bearing ancestor only to later evolve without tusks while retaining their semi-burrowing lifestyle [14].

Owing to there being no mt genomes of Euthyplociidae, Ichthybotidae, Behningiidae, or Palingeniidae released on the NCBI site and even some families like Euthyplociidae not being distributed in China [111], this study mainly focused on Potamanthidae, Ephemeridae, and Polymitarcyidae for discussion. For future research, a concerted effort by domestic and international experts is also required to expand the mt genomes of these families and establish a more robust phylogenetic relationship for burrowing mayflies.

### 4.3. The Evolutional Time of Potamanthidae

In this study, our analysis showed results that confirmed that all families of Potamanthidae originated during the Mesozoic Era, which is consistent with Tong et al. and García-Girón et al. [26,29,40], and Potamanthidae originated in the mid-Mesozoic Era, with two genera diverging in the late Mesozoic Era. The period of the Mesozoic Era has always been considered as the time of maximum growth for mayflies and occurred along with glacial activities and the appearance of large aquatic plants and angiosperms [35,112,113]. In addition, Lord discovered the presence of algae in the stomach contents of larvae during dissection, indicating that this taxon fed on algae, consistent with the appearance of calcium algae and diatoms in the Mesozoic Era that could also provide an optimal environment for the origin of the Potamanthidae [114,115]. The known fossils of Potamanthidae were speculated to have originated in the Cretaceous period, and the formation of these fossils was within the time interval for the inferred origin of Potamanthidae, as determined by this study [30,31,38,116]. In addition, Siphluriscidae diverged at 193.96 Mya (95% HPD, 169.80–226.13 Mya) in the Mesozoic Jurassic period. This is consistent with the fossil (belonging to Siphluriscidae) formation time in the Mesozoic Jurassic period as described by Zhou et al. [117]. In addition, a description of *Jurassonurus amoenus* (Siphluriscidae) from the Jurassic period on Jiulong Mountain (Inner Mongolia, China) was described by Huang et al. [96]. That study predicted the origin time of the Baetidae to be 115.31 Mya (95% HPD, 91.96–135.86 Mya), which was consistent with the earliest members of Baetidae that were discovered in Early Cretaceous amber from Lebanon [34]. The above fossil evidence can provide support for the results of the current research.

### 4.4. Positive Selection Analysis

Research has already demonstrated that the 13 PCGs within the mt genome may be subject to positive selection under the influence of low temperatures [57,118]. In the present study, we found that the 258th site in ATP8 and the 2265th site in ND2 had higher evolutionary rates using the site model, which utilizes various site class-specific models and assumes that all branches have the same ω ratio but differ among sites in alignment [100]. The results suggest that the entire Potamanthidae family may have undergone adaptive evolutionary selection for the 13 PCGs.

ND2 and ATP8 belong to complex I and complex V, respectively, of the mitochondria electron transport chain. Complex I of the mt genome contains seven PCGs (ND1–ND4, ND4L, ND5, and ND6) and contributes at least one third of the total ATP production by cells [119]. Within complex I, ND2 also plays an important role in the structure of the intramembrane arm and peripheral arm [120]. ATP8 is one of the subunits of ATP synthase (complex V) which is essential for the electron transport chain that provides ATP to organisms [121]. Xu et al. confirmed that the ND2 gene was also under positive selection in Heptageniidae living in long-term cold environments [57]. Furthermore, apart from insects, Hong et al. found that the foreground branch of *Hyla* (Anura, Hylidae) and *Dryophytes* (Anura, Hylidae) has been persistently exposed to cold environments had five positive sites in Cyt b, ND3, ND4, and ND5 [118]. It has also been suggested that different species may adopt different strategies in extreme environments. For Potamanthidae, the rapid evolutionary rate of ATP8 and ND2 may be a strategy of adaptation to extreme environments.

We found that no branch or site were under positive selection in the branch model or branch-site model. In general, a limited number of sites were under positive selection, and the evolutionary time was always short. Thus, the signal of positive selection is overshadowed by the ongoing effect of negative selection occurring at the majority of sites in the gene sequence. In addition, a short positive selection was often followed by a long washout selection, which complicates the identification of selection mechanisms [122]. This could also be the reason for the absence of positive selection sites in this study. Negative selection was found in *R. coreanus.* In order to maintain mitochondrial function, organisms often restrict activities that are not conducive to energy production by removing harmful mutations through negative selection [44], and this may also suggest that negative selection in *R. coreanus* may be a way to constrain the production of ATP to adapt to cold environments.

## 5. Conclusions

In our study, we sequenced seven mt genomes of Potamanthidae, and these were consistent with the characteristics of insect mt genomes. In all seven mt genomes, COX1 used CGA as its start codon, and the absence of the DHU arm of S1 also occurred in all seven species. These characteristics also appeared in the research of other scholars. In addition, we recovered the relationship in which Potamanthidae was a sister clade to Ephemeridae + Polymitarcyidae, with a high posterior probability and bootstrap value. Based on five fossil calibration points, we calculated the divergence times within Ephemeroptera, and the results showed that Potamanthidae originated at 90.44 Mya (95% HPD, 62.80–121.74 Mya). Then, *Rhoenanthus* and *Potamanthus* genera diverged at 64.77 Mya (95% HPD, 43.82–88.68 Mya) in the late Pliocene Epoch or early Miocene Epoch. In the analysis of positive selection, we found that *R. coreanus* was under negative selection. Finally, we also found the ATP8 and ND2 had the highest evolutionary rate in Potamanthidae, which may be a strategy of adaptation to extreme environments.

## Figures and Tables

**Table 1:** Information about samples used in this study and their NCBI GenBank accession numbers.

Specimen No.	Species	Genera	Sampling Localities	Accession No.
01YNAS	*Rhoenanthus obscurus*	*Rhoenanthus*	Mengla, Yunnan	PP473793
LNDD4	*Rhoenanthus coreanus*	*Rhoenanthus*	Dandong, Liaoning	PP473799
02JHGD	*Potamanthus* sp. 02JHGD	*Potamanthus*	Jinhua, Zhejiang	PP473796
02WZ10	*Potamanthus* sp. 02WZ10	*Potamanthus*	Wenzhou, Zhejiang	PP473797
08HH02	*Potamanthus* sp. 08HH02	*Potamanthus*	Jianou, Fujian	PP473798
HHFK100	*Potamanthus longitibius*	*Potamanthus*	Shangrao, Jiangxi	PP473794
LNDD5	*Potamanthus luteus*	*Potamanthus*	Dandong, Liaoning	PP473795

**Table 2:** Partition schemes and best evolutionary models obtained from PartionFinder 2.2.1.

Subset	Subset Partitions	Best Model
Partition_1	ND4L_pos1, 16S_pos1, 12S_pos1, 12_pos2, 12_pos3, 16S_pos2	TVM + I + G
Partition_2	COX3_pos1, COX2_pos1, Cyt b_pos1, ATP6_pos1	GTR + I + G
Partition_3	COX1_pos2, CYTB_pos2, COX2_pos2, ATP6_pos2, COX3_pos2	TVM + I + G
Partition_4	COX3_pos3, ATP6_pos3	TRN + I + G
Partition_5	ATP8_pos1, ND2_pos1, ND3_pos1, ND6_pos1	TVM + I + G
Partition_6	ATP8_pos2, ND2_pos2, ND6_pos2, ND3_pos2	GTR + I + G
Partition_7	ND6_pos3, ATP8_pos3	HKY + I + G
Partition_8	COX1_pos1	GTR + I + G
Partition_9	COX1_pos3	TRN + I + G
Partition_10	CYTB_pos3, ND3_pos3, COX2_pos3	TIM + I + G
Partition_11	ND5_pos1, ND1_pos1, ND4_pos1	GTR + I + G
Partition_12	ND5_pos2, ND4_pos2, ND4L_pos2, ND1_pos2	GTR + I + G
Partition_13	ND1_pos3, ND4_pos3, ND5_pos3	GTR + G
Partition_14	ND2_pos3	TRN + G
Partition_15	ND4L_pos3	TRN + I + G

**Table 3:** The AT content, CG skew and AT skew of each species in its mt genome, including 13 PCGs on the heavy strand and 2 rRNAs on the light strand.

Species	mt Genome			PCGs			rRNA		
	A+T%	AT-K	CG-K	A+T%	AT-K	CG-K	A+T%	AT-K	CG-K
*Rhoenanthus coreanus*	70.2	−0.048	−0.219	67.9	−0.196	−0.163	72.2	0.058	0.296
*Rhoenanthus obscurus*	69.3	−0.048	−0.233	65.6	−0.186	−0.191	70.9	0.060	0.304
*Potamanthus* sp. 02JHGD	66.2	0	−0.130	64.4	−0.176	−0.129	69.5	0.022	0.294
*Potamanthus* sp. 02WZ10	67.8	−0.002	−0.242	64.9	−0.159	−0.191	70.8	0.012	0.289
*Potamanthus* sp. 08HH02	67.4	−0.012	−0.211	64.4	−0.176	−0.163	70.2	0.020	0.281
*Potamanthus longitibius*	66.6	−0.018	−0.179	64.6	−0.175	−0.140	69.5	0.022	0.281
*Potamanthus luteus*	69.7	−0.036	−0.228	66.6	−0.170	−0.176	73.2	0.025	0.301

## Data Availability

Data to support this study are available from the National Center for Biotechnology Information (https://www.ncbi.nlm.nih.gov) (accessed on 20 March 2024). The GenBank numbers are PP473793–PP473799.

## Supplementary Materials

The following supporting information can be downloaded at. https://www.mdpi.com/article/10.3390/insects15050357/s1. Figure S1: The secondary structures of seven species. Figure S2: The results for FcML analysis, where Cluster 1 = Potamanthidae + (Ephemeridae + Polymitarcyidae); Cluster 2 = Caenidae; Cluster 3 = Neoephemeridae; and Cluster 4 = Teloganodidae + Baetidae. Table S1: Sources of mitochondrial genomic data other than those in this study. Table S2: Location of features in the seven mt genomes. Table S3: Divergence times for nodes or clades in Ephemeroptera. All estimates are represented in terms of millions of years ago (Mya), and “&” represents the relationship of two branches. Table S4: The resulting site model when we used the topology structure with all species of Potamanthidae. Table S5: The resulting branch model when we used two different topology structures with two different foreground branches. Table S6: The results of the branch-site model when we used two different topology structures with two different foreground branches.
