# Supplementary material for: Comparative Mitogenome of Phylogenetic Relationships and Divergence Time Analysis within Potamanthidae (Insecta: Ephemeroptera)
Source: Insects. 2024 May 15;15(5):357. doi: 10.3390/insects15050357 (PMC11122660; doi:10.3390/insects15050357)
Supplement: Supplementary file 1 [file insects-15-00357-s001.zip › Table S1.pdf]

Table S1. Sources of mitochondrial genomic data other than in this study.

S/N	Species	Family	Accession No.	Reference
1	*Ameletus* sp. 1 MT-2014	Ameletidae	KM244682	[57]
2	*Cloeon* dipterum	Baetidae	MW149047	[58]
3	*Baetis* sp. PC-2010	Baetidae	GU936204	[59]
4	*Takobia yixiani*	Baetidae	GU479735	[59]
5	*Nigrobaetis niger*	Baetidae	MT483692	Direct Submission
6	*Procloeon bifidum*	Baetidae	MT483677	Direct Submission
7	*Caenis* sp. JYZ-2020	Caenidae	MN356096	[60]
8	*Caenis* sp. JYZ-2018	Caenidae	MG910499	[61]
9	*Caenis horaria*	Caenidae	MT622520	Direct Submission
10	*Caenis robusta*	Caenidae	MT584126	Direct Submission
11	*Caenis robusta*	Caenidae	MT628575	Direct Submission
12	*Caenis pycnacantha*	Caenidae	GQ502451	Unpublished
13	*Torleya mikali*	Ephemerellidae	MT535766	[62]
14	*Cincticostella fusca*	Ephemerellidae	MT535767	[62]
15	*Torleya nepalica*	Ephemerellidae	MT274132	[68]
16	*Torleya grandifrceps*	Ephemerellidae	MT274131	[68]
17	*Serratella zapekinae*	Ephemerellidae	MT274130	[68]
18	*Ephemerella* sp. Yunnan-2018	Ephemerellidae	MT274127	[68]
19	*Serratella* sp. Liaoning-2019	Ephemerellidae	MT274128	[68]
20	*Serratella ignita*	Ephemerellidae	MT628582	Direct Submission
21	*Ephemerella* sp. MT-2014	Ephemerellidae	KM244691	[57]
22	*Vietnamella sinensis*	Viemamellidae	OK265111	[33]
23	*Ephemera serica*	Ephemerellidae	OK018134	Direct Submission
24	*Hexagenia rigida*	Ephemerellidae	OL678102	[21]
25	*Ephemera orientalis*	Ephemerellidae	EU591678	[64]
26	*Ephemera* sp. XL-2019	Ephemerellidae	MK951659	[70]
27	Heptagenidae sp. YW03BF02	Heptagenidae	MK642300	[44]
28	*Epeorus* sp. 06 ZXM-2022a	Heptagenidae	OK495706	[65]
29	*Epeorus* sp. 05 ZXM-2022a	Heptagenidae	OK495705	[65]
30	*Epeorus psi*	Heptagenidae	OK495704	[65]
31	*Epeorus dayongensis*	Heptagenidae	OK495703	[65]
32	*Epeorus* sp. 04 ZXM-2022a	Heptagenidae	OK495702	[65]
33	*Epeorus herklotsi*	Heptagenidae	OK495701	[65]
34	*Epeorus* sp. 03 ZXM-2022a	Heptagenidae	OK495700	[65]
35	*Epeorus alexandri*	Heptagenidae	OK495699	[65]
36	*Epeorus* sp. 02 ZXM-2022a	Heptagenidae	OK495698	[65]
37	*Epeorus rhithralis*	Heptagenidae	OK495697	[65]
38	*Epeorus bispinosus*	Heptagenidae	OK495696	[65]
39	*Epeorus aculeatus*	Heptagenidae	OK495695	[65]
40	*Epeorus* sp. 01 ZXM-2022a	Heptagenidae	OK495694	[65]
41	*Epeorus unispinosus*	Heptagenidae	OK495693	[65]
42	*Cinygmina* sp. 07BF96	Heptagenidae	MW450878	[44]
43	*Stenonema femoratum*	Heptagenidae	MK642306	[44]
44	*Stenacron interpunctatum*	Heptagenidae	MK642305	[44]
45	*Maccaffertium vicarium*	Heptagenidae	MK642304	[44]
46	*Leucrocuta aphrodite*	Heptagenidae	MK642301	[44]
47	*Afronurus yixingensis*	Heptagenidae	MK642297	[44]
48	*Cinygmina* sp. 1 YW01BF06	Heptagenidae	MK642295	[44]
49	*Afronurus levis*	Heptagenidae	OQ863614	Direct Submission
50	*Rhithrogena germanica*	Heptagenidae	MT584121	Direct Submission
51	*Electrogena lateralis*	Heptagenidae	MT874480	Direct Submission
52	*Paegniodes cupulatus*	Heptagenidae	MW381300	[62]
53	*Notacanthurus lamellosus*	Heptagenidae	MW381298	[62]

54	*Parafronurus youi*	Heptagenidae	EU349015	[36]
55	*Isonychia kiangsinensis*	Isonychidae	MH119135	[66]
56	*Isonychia ignota*	Isonychidae	HM143892	Unpublished
57	*Choroterpes yixingensis*	Leptophlebidae	MW717290	[67]
58	*Deleatidium vernale*	Leptophlebidae	OR414023	Direct Submission
59	*Leptophlebia vespertina*	Leptophlebidae	MT622515	Direct Submission
60	*Leptophlebia marginata*	Leptophlebidae	MT622514	Direct Submission
61	*Habrophlebiodes zjiensis*	Leptophlebidae	GU936203	[59]
62	*Potamanthellus edmundsi*	Neoephemeridae	OK272543	Direct Submission
63	*Neoephemera projecta*	Neoephemeridae	OK272542	Direct Submission
64	*Ephoron yunnanensis*	Polymitarcyidae	MF352159	[68]
65	*Potamanthus kwangsiensis*	Potamanthidae	MF352158	[68]
66	*Rhoenanthus* sp.	Potamanthidae	MF352145	[68]
67	*Potamanthus* sp. MT-2014	Potamanthidae	KM244674	[57]
68	*Siphlonurus immanis*	Siphlonuridae	FJ606783	Unpublished
69	*Siphlonurus aestivalis*	Siphlonuridae	MT862395	Direct Submission
70	*Siphlonurus* sp. MT-2014	Siphlonuridae	KM244684	[57]
71	*Siphluriscus chinensis*	Siphluriscidae	HQ875717	[69]
72	*Siphluriscus* sp. I JZ-2022	Siphluriscidae	ON729391	[20]
73	Teloganodidae sp.	Teloganodidae	KM244703	[57]
