# Supplementary material for: Comparative Mitogenome of Phylogenetic Relationships and Divergence Time Analysis within Potamanthidae (Insecta: Ephemeroptera)
Source: Insects. 2024 May 15;15(5):357. doi: 10.3390/insects15050357 (PMC11122660; doi:10.3390/insects15050357)
Supplement: Supplementary file 1 [file insects-15-00357-s001.zip › Table S3.pdf]

Table S3. Divergence times for nodes/clades in the Ephemeroptera. All estimates are represented in millions of years ago (Mya), and "&" represents the relationship of two branches.

Nodes/Clades	Mean Divergence-time (Mya)	95 % HPD Range (Mya)
Ephemeridae & Polymitarcyidae	70.70	47.67-95.72
Potamanthidae & (Ephemeridae + Polymitarcyidae)	64.77	62.80-121.74
Caenidae & Neoephemeridae	94.75	69.97-124.55
(Potamanthidae + (Ephemeridae + Polymitarcyidae)) & (Caenidae + Neoephemeridae)	115.13	86.11-145.70
Baetidae & Teloganodidae	115.31	91.96-135.86
Ephemerellidae & Viemamellidae	98.80	98.17-99.41
(Baetidae + Teloganodidae) & (Ephemerellidae + Viemamellidae)	133.91	113.04-152.75
((Baetidae + Teloganodidae) + (Ephemerellidae + Viemamellidae)) & Leptophlebiidae	141.26	122.21-161.46
((Potamanthidae + (Ephemeridae + Polymitarcyidae)) + (Caenidae + Neoephemeridae)) & ((Baetidae + Teloganodidae) + (Ephemerellidae + Viemamellidae) + Leptophlebiidae)	149.12	129.69-168.36
(((Potamanthidae + (Ephemeridae + Polymitarcyidae)) + (Caenidae + Neoephemeridae)) + ((Baetidae + Teloganodidae) + (Ephemerellidae + Viemamellidae) + Leptophlebiidae)) & Heptageniidae	162.77	146.40-180.14
Ameletidae & Siphlonuridae	159.77	158.98-160.58
((((Potamanthidae + (Ephemeridae + Polymitarcyidae)) + (Caenidae + Neoephemeridae)) + (((Baetidae + Teloganodidae) + (Ephemerellidae + Viemamellidae) + Leptophlebiidae)) + Heptageniidae) & (Ameletidae + Siphlonuridae)	171.82	161.98-185.62
(((((Potamanthidae + (Ephemeridae + Polymitarcyidae)) + (Caenidae + Neoephemeridae)) + ((Baetidae + Teloganodidae) + (Ephemerellidae + Viemamellidae) + Leptophlebiidae)) + Heptageniidae) + (Ameletidae + Siphlonuridae)) & Isonychiidae	183.44	167.02-202.64
