# Supplementary material for: Comparative Mitogenome of Phylogenetic Relationships and Divergence Time Analysis within Potamanthidae (Insecta: Ephemeroptera)
Source: Insects. 2024 May 15;15(5):357. doi: 10.3390/insects15050357 (PMC11122660; doi:10.3390/insects15050357)
Supplement: Supplementary file 1 [file insects-15-00357-s001.zip › Table S4.pdf]

Table S4 The resulting site model when we used the topology structure with all species of Potamanthidae.

Model	Ln L	Estimates of parameters				Model compared	LRT P-value	Positive sites
M3	-45424.794432	p	0.86440	0.13560	0.00000			
		ω	0.00722	0.22051	189.32568	M0 vs. M3	0.000000000	
M0	-46213.183935	ω0		0.03054				
M2a	-45706.596285	p	0.95638	0.04362	0.00000			
		ω	0.01970	1.00000	27.85669	M1a vs. M2a	0.999940002	
M1a	-45706.596225	p	0.95638	0.04362				
		ω	0.01975	1.00000				
M8	-45428.898456	p0= 0.98158	p= 0.03045	q= 0.24021				
		(p1= 0.01842)	ω= 1.00000					
M7	-45526.861210	p= 0.03218		q=0.23816		M7 vs.M8	0.000000000	256 S 0.625,258 R 0.984*,264 S 0.819,265 R 0.843,268 S 0.637,1834 Q 0.637,2040 D 0.579,2043 K 0.695,2044 F 0.796,2261 L 0.597,2262 T 0.812,2265 S 0.936,2311 C 0.743,2378 F 0.756,2828 R 0.524,3137 I 0.568,3141 W 0.871,3193 L 0.558,3198 D 0.566,3618 L 0.799,3621 R 0.544,3622 I 0.587,3643 F 0.696,3644 L 0.837,3646 S 0.677,3648 L 0.866
M8a	-45428.898454	p0=0.98158	p=0.03006	q=0.23568		M8a vs.M8	0.998404232	
		(p1= 0.01842)		ω= 1.00000				
