# Supplementary material for: Comparative Mitogenome of Phylogenetic Relationships and Divergence Time Analysis within Potamanthidae (Insecta: Ephemeroptera)
Source: Insects. 2024 May 15;15(5):357. doi: 10.3390/insects15050357 (PMC11122660; doi:10.3390/insects15050357)
Supplement: Supplementary file 1 [file insects-15-00357-s001.zip › Table S5.pdf]

Table S5. The resulting branch model when we used two different topology structures with two different foreground branches, respectively.

Foreground Branch	Model	np	Ln L	Estimates of parameters			Model compared	LRT P-value	Omega for Foreground Branch
R. coreanus	Two ratio Model 2	19	-41032.577533	ω:	ω 0= 0.03065	ω 1= 0.02035	Two ratio Model 2 vs. Model 0	0.002391843	ω 1= 0.02035
	Model 0	18	-41037.188303	ω=	0.02924				
P. luteus	Two ratio Model 2	19	-42114.968291	ω:	ω 0= 0.03041	ω 1=0.02982	Two ratio Model 2 vs. Model 0	0.858612719	
	Model 0	18	-42114.984158	ω=	0.03032				
