# Supplementary material for: Comparative Mitogenome of Phylogenetic Relationships and Divergence Time Analysis within Potamanthidae (Insecta: Ephemeroptera)
Source: Insects. 2024 May 15;15(5):357. doi: 10.3390/insects15050357 (PMC11122660; doi:10.3390/insects15050357)
Supplement: Supplementary file 1 [file insects-15-00357-s001.zip › Table S6.pdf]

Table S6. The result of branch-site model when we used two different topology structures with two different foreground branches, respectively.

Foreground Branch	Model	np	Ln L	Estimates of parameters					Model compared	LRT P-value	Positive sites
R. coreanus	MA	21	-40669.658913	Site class	0	1	2a	2b	Model A vs. Model A null	0.345227577	218 S 0.942,762 I 0.737
				f	0.96182	0.03745	0.00071	0.00003			
				ω0	0.01892	1.00000	0.01890	1.00000			
				ω1	0.01890	1.00000	30.88025	30.88025			
	Model A null	20	-40670.104374	1							
P. luteus	MA	21	-41634.650166	Site class	0	1	2a	2b	Model A vs. Model A null	0.103961041	13 T 0.566,347 L 0.877,420 F 0.962*,546 S 0.599,682 S 0.861,713 N 0.782,715 S 0.506,884 G 0.778,892 N 0.747,1374 P 0.500,1417 S 0.832,1454 V 0.596,1583 I 0.657,1702 P 0.849,1712 L 0.967*,1715 I 0.636,2091 A 0.852,2282 A 0.575,2464 N 0.914,3462 F 0.685,3466 S 0.878
				f	0.94670	0.04084	0.01195	0.00052			
				ω0	0.01819	1.00000	0.01819	1.00000			
				ω1	0.01819	1.00000	29.57838	29.57838			
	Model A null	20	-41633.328315	1							
