# Supplementary figures and images for: Comparative Mitogenome of Phylogenetic Relationships and Divergence Time Analysis within Potamanthidae (Insecta: Ephemeroptera)
Source: Insects. 2024 May 15;15(5):357. doi: 10.3390/insects15050357 (PMC11122660; doi:10.3390/insects15050357)

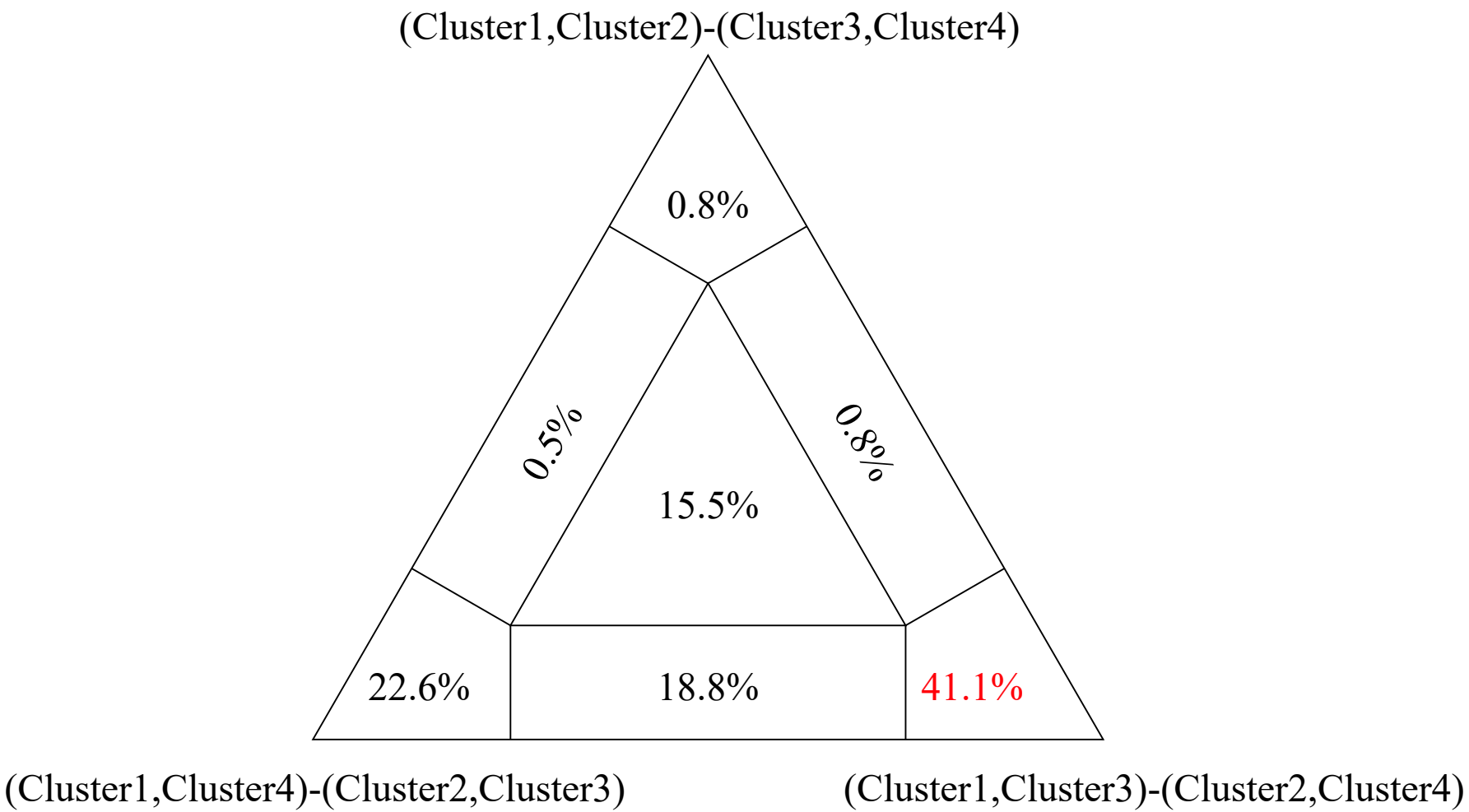

Supplement: Supplementary file 1 [file insects-15-00357-s001.zip › Figure S2.pdf]
