# Supplementary material for: Comparative Mitogenome of Phylogenetic Relationships and Divergence Time Analysis within Potamanthidae (Insecta: Ephemeroptera)
Source: Insects. 2024 May 15;15(5):357. doi: 10.3390/insects15050357 (PMC11122660; doi:10.3390/insects15050357)
Supplement: Supplementary file 1 [file Figure S1.pdf › Figure S1.pdf]

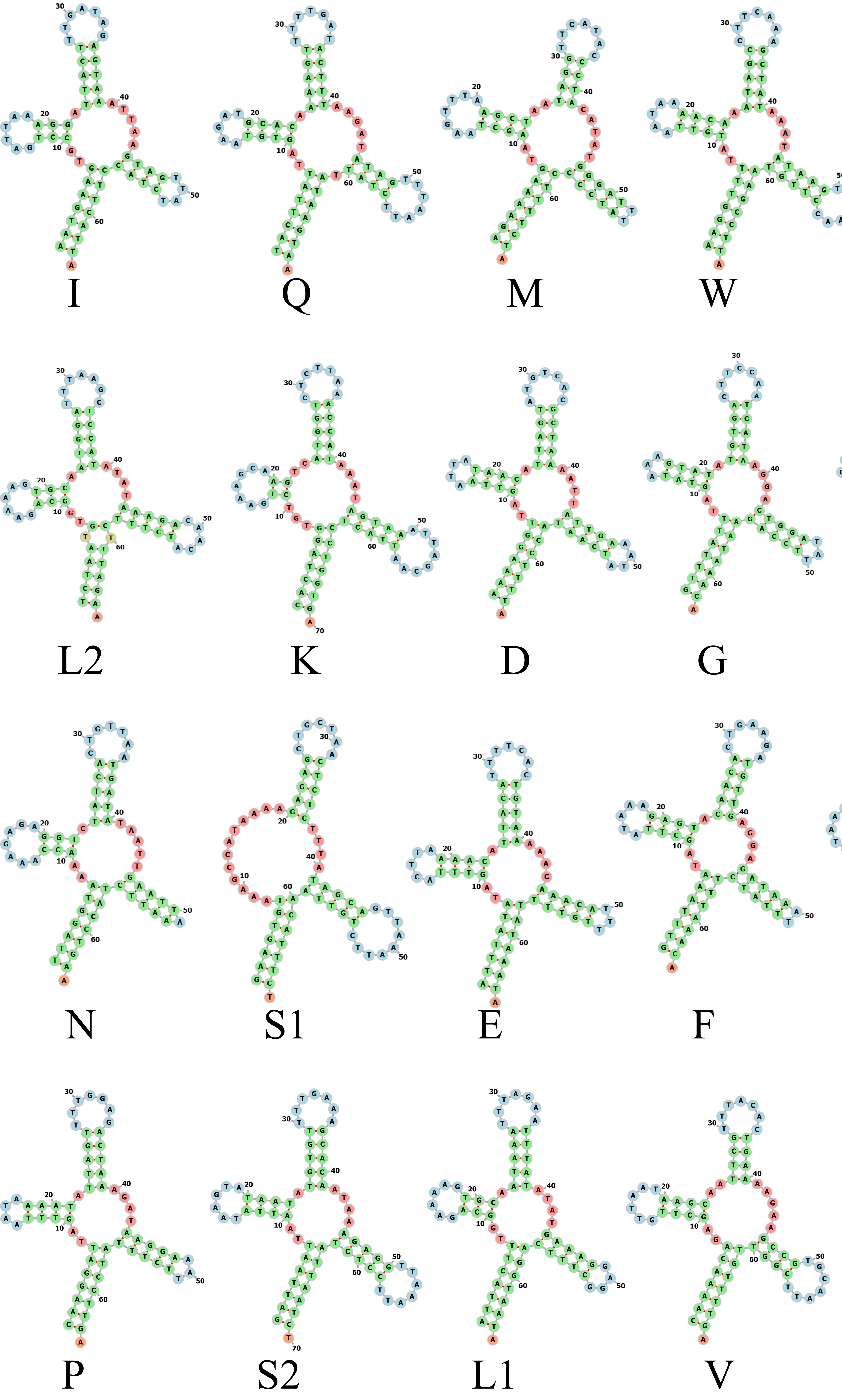

(A) ***Rhoenanthus obscurus***

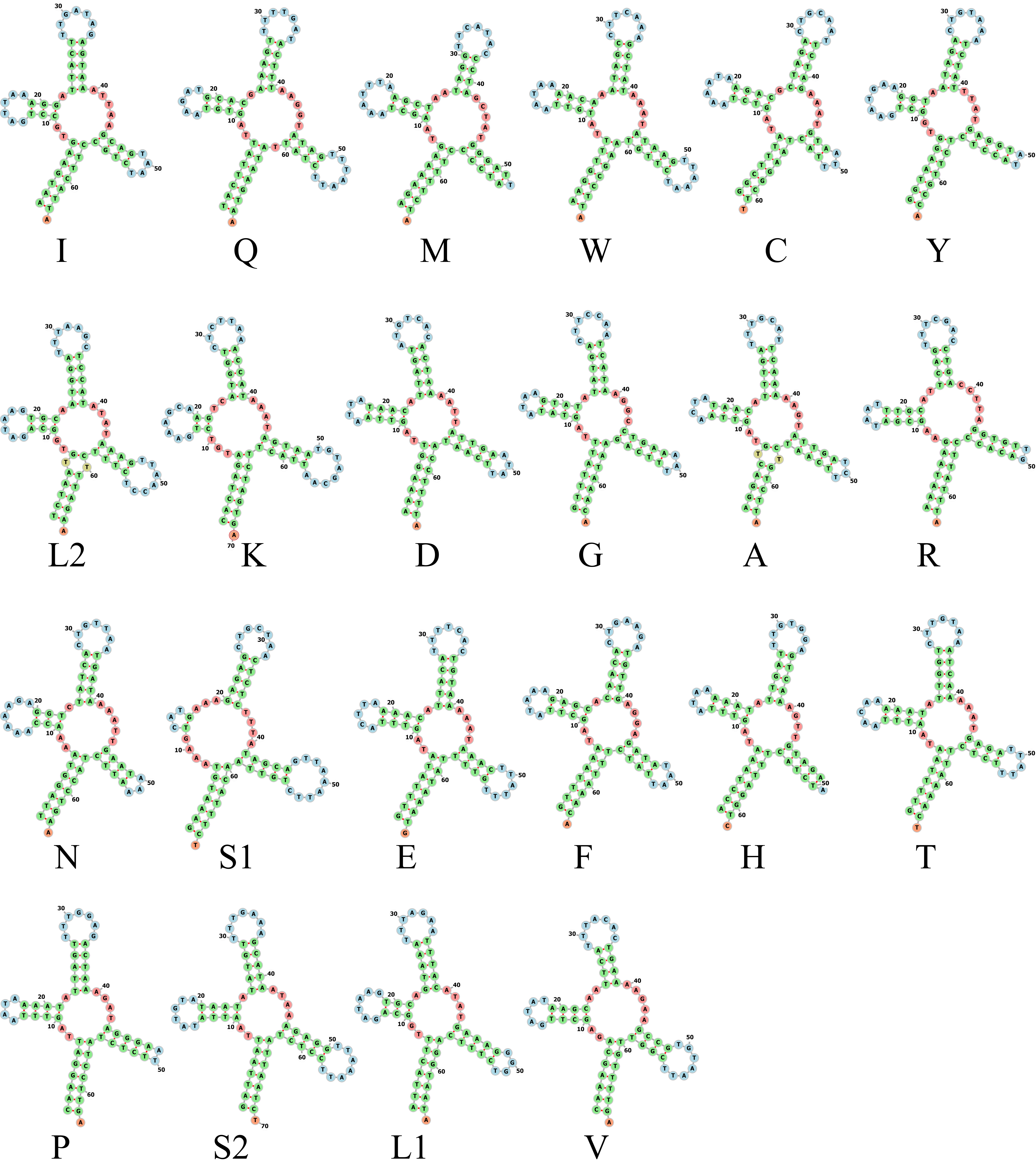

(B) *Rhoenanthus coreanus*

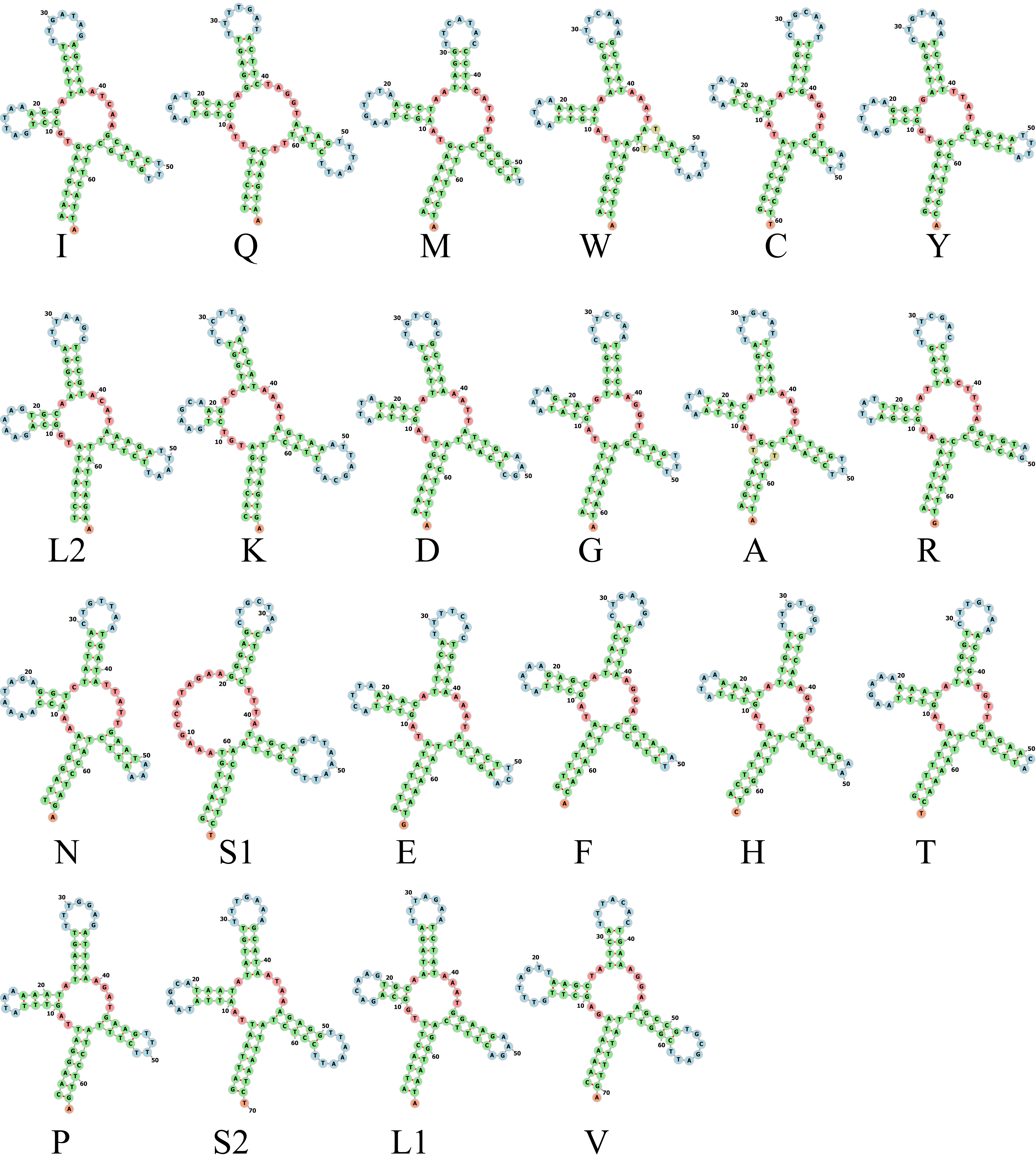

(C) *Potamanthus* sp. 02JHGD

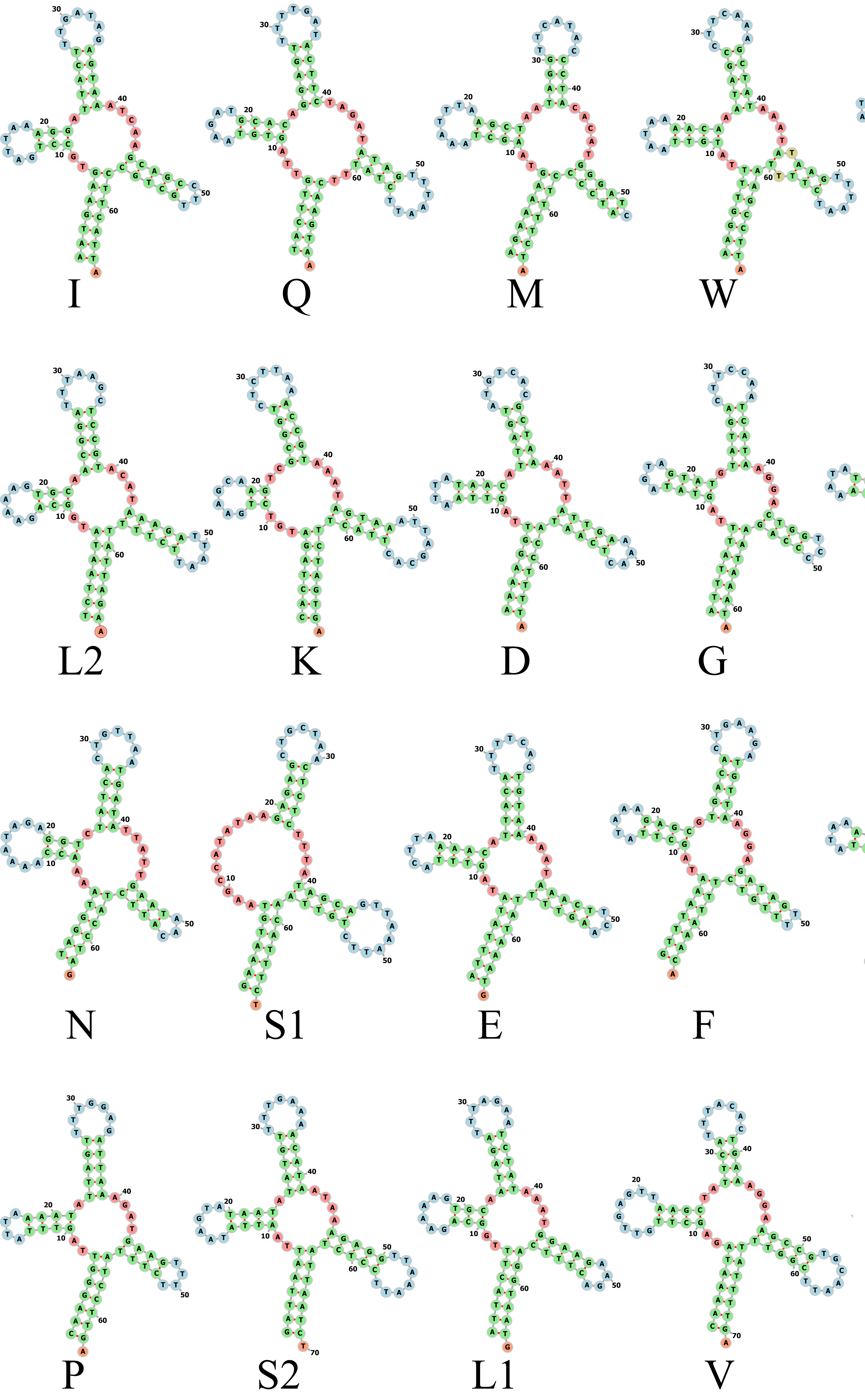

(D) *Potamanthus* sp. 02WZ10

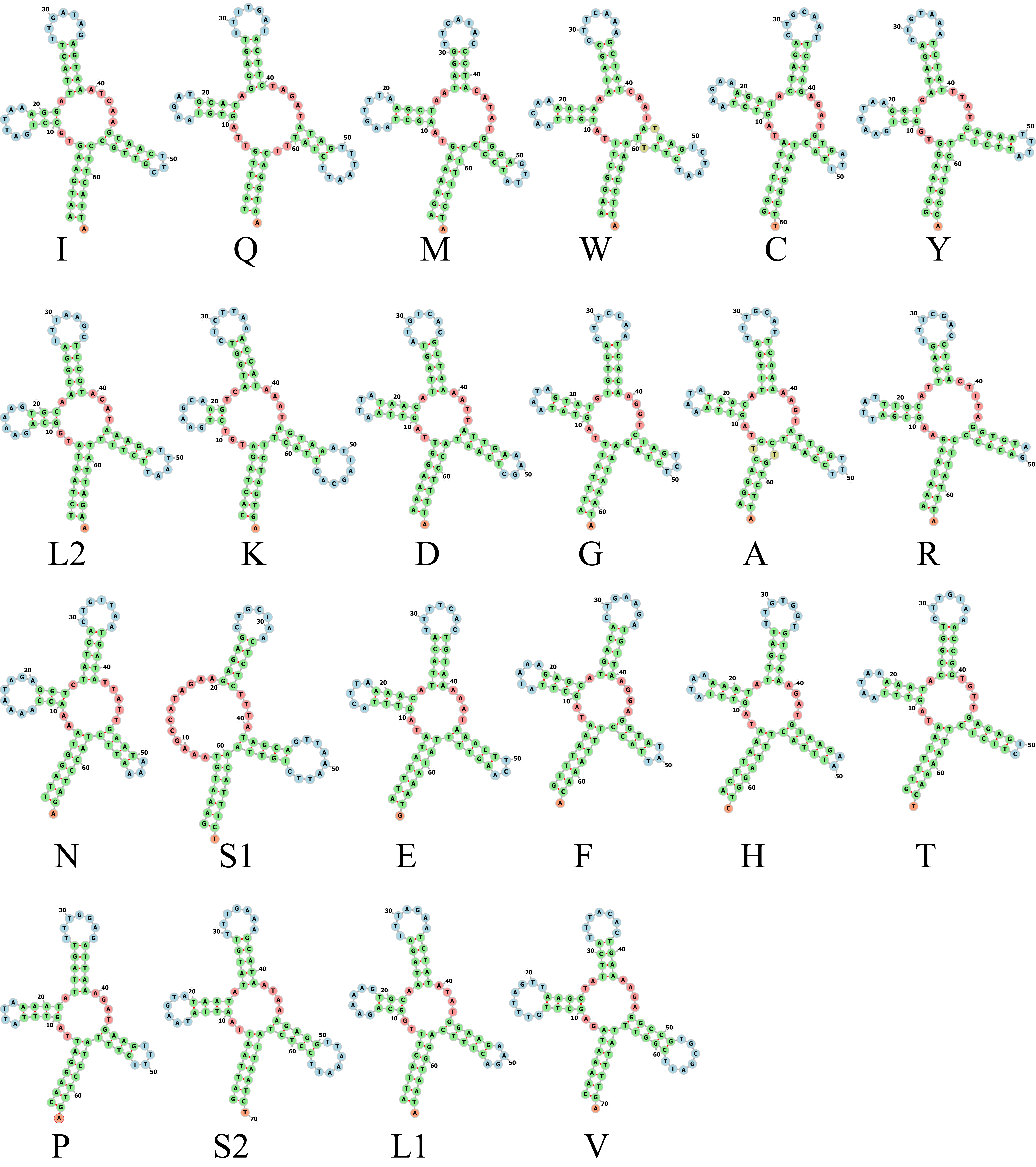

(E) *Potamanthus* sp. 08HH02

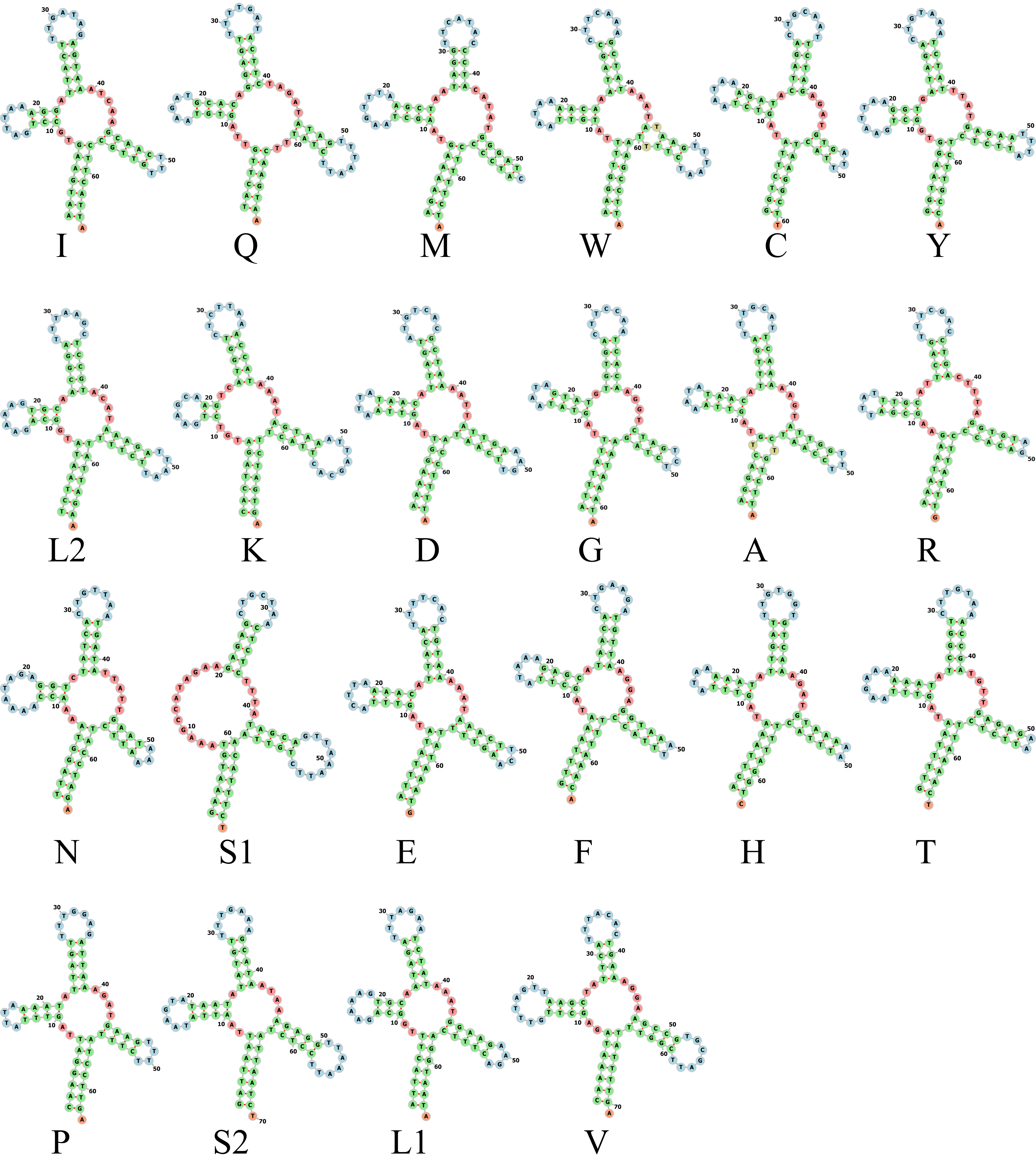

(F) ***Potamanthus longitibius***

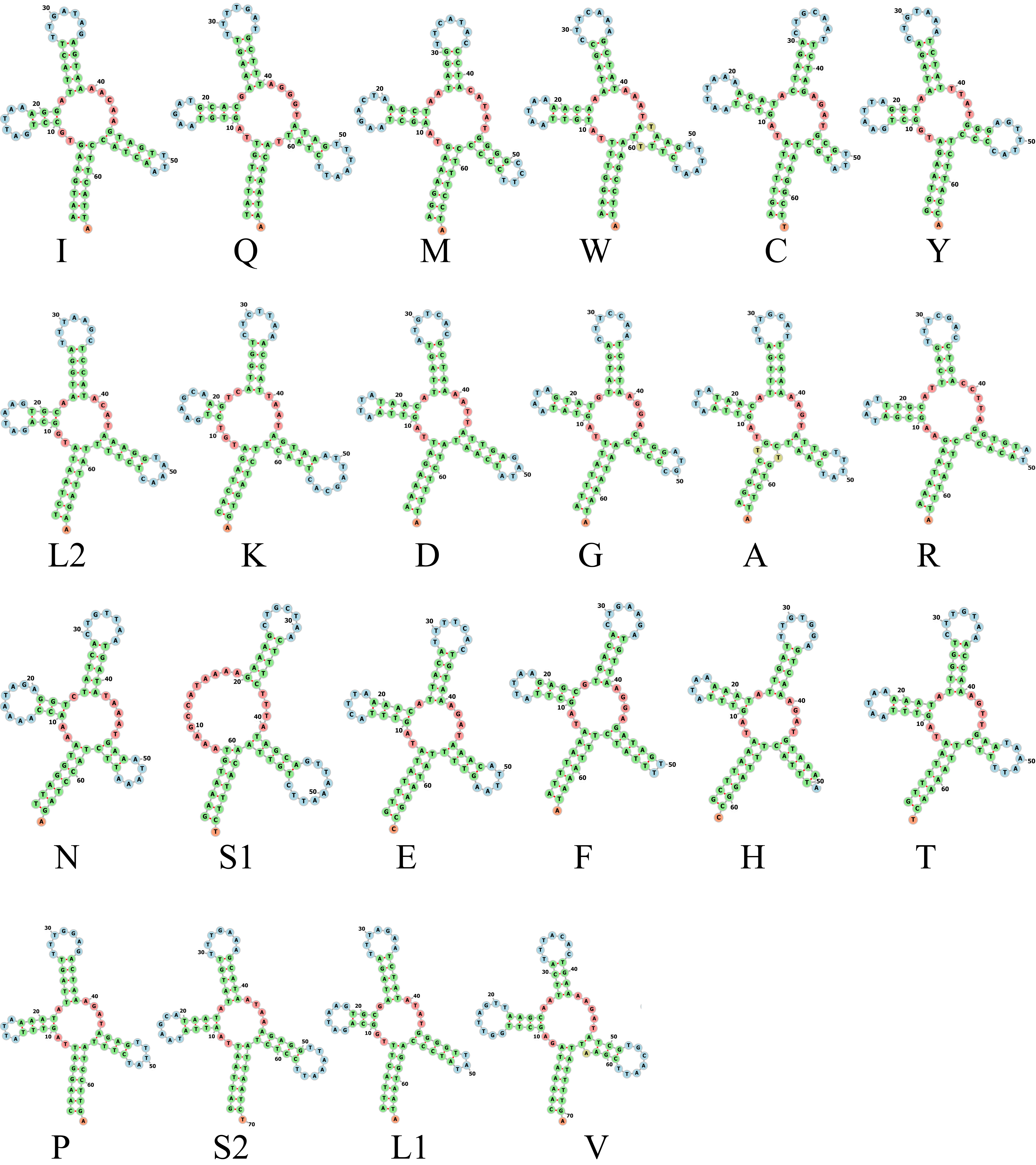

(G) *Potamanthus luteus*
